# Chronic gut inflammation potentiates place aversion in intrinsic and conditioned contexts

**DOI:** 10.1016/j.bbih.2026.101383

**Published:** 2026-10-08

**Authors:** B. Cej, K. Beekman, C.E. Matisz, T. Haight, A. Zovoilis, R.J. McDonald, A.J. Gruber

**Affiliations:** a Department of Cell Biology and Anatomy, Cumming School of Medicine, University of Calgary, 3330 Hospital Drive NW, Calgary, AB, T2N 4N1, Canada; b Alberta Children's Hospital Research Institute, University of Calgary, 3330 Hospital Drive NW, Calgary, AB, T2N 4N1, Canada; c University of Lethbridge, Canadian Centre for Behavioural Neuroscience, 4401 University Drive W, Lethbridge, AB, T1K 3M4, Canada; d Department of Biochemistry and Medical Genetics, University of Manitoba, 745 Bannatyne Avenue, Winnipeg, MB, R3E 0J9, Canada; e Paul Albrechtsen Research Institute, CancerCare Manitoba, 675 McDermot Avenue, Winnipeg, MB, R3E 0V9, Canada

**Keywords:** Colitis, Fear extinction, Nucleus accumbens, Anterior cingulate cortex, Anxiety, Aversion

## Abstract

Chronic inflammation in the body evokes behavioural changes that differ from changes following acute inflammation. These include anxio-depressive-like behaviours, which may serve an adaptive function to mitigate increased existential risk when animals are ill or injured. We test here if chronic gut inflammation induced by dextran sodium sulfate (DSS) promotes contextually-specific avoidance in mice. DSS-treated mice and vehicle-treated mice avoided a testing chamber that was previously paired with a mild foot shock relative to an unpaired contextually-distinct chamber. When animals were tested a second time one week later, mice treated with DSS maintained the same level of aversion to the paired context, whereas vehicle-treated mice reduced their aversion. These results suggest that DSS-treated animals had prolonged aversion to a context previously paired with a negative stimulus. In an open field test, DSS-treated mice exhibited increased aversion to the centre of the arena. Centre aversion correlated with the severity of gut inflammation. These results suggest that chronic gut inflammation enhanced aversion to potentially threatening contexts, both intrinsic and learned. DSS-treated mice also exhibited heterogeneous changes in gene transcript expression in neural structures known to play a role in contextual place avoidance. Expression of the proinflammatory cytokine *Il1b* mRNA was increased in the nucleus accumbens, and expression of the antioxidant regulator *Nrf2* was increased in the anterior cingulate cortex. These transcriptional changes co-occur with the observed behavioural phenotypes and support a growing body of evidence indicating that chronic gut inflammation alters the brain to promote anxiety-like phenomena, which may help account for the high comorbidity of anxiety diseases in people with chronic somatic inflammation.

## Highlights

• Gut inflammation doesn't impair contextual place aversion.
• Gut inflammation prolongs aversion to a context previously paired with mild foot-shock.
• Mice with gut inflammation spend less time in the center of an open field.
• Gut inflammation is associated with increased IL-1b in the nucleus accumbens.
• Gut Inflammation is associated with increased Nrf2 in the anterior cingulate cortex.

## 1 Introduction

Inflammation in the body produces a well-defined suite of sickness behaviours (D'Mello and Swain, 2016). Most studies assessing sickness behaviours have focused on relatively brief bouts of inflammation lasting a few hours (e.g. injection of bacterial or viral products), to 5 days (e.g. acute colitis using the dextran sodium sulfate model). Much less is known about the effects of chronic inflammation, which evokes a different set of behavioural changes than acute inflammation (Matisz et al., 2020). In humans, chronic inflammation is highly co-morbid with anxiety and depression. It is estimated that patients with inflammatory bowel diseases (IBD) possess anxiety and depression at rates 1.5 times greater than the general population (Barberio et al., 2021). Rates of these mental disorders are highest during active disease but are also significantly elevated during periods of remission. Clinical evidence suggests that chronic neuroinflammation plays a central role in the mentation changes (anxiety, depression, cognitive dysfunction) that accompany IBD. Although these findings are generally consistent with studies of inflammation in animals, few animal studies assess how chronic inflammation affects brain and behaviour.

When the brain senses inflammation, indicating illness or injury, the resulting changes to behaviour appear to improve fitness in ethological context, for instance by reducing risk exposures (Hart, 1988). We further propose that injury/illness increases risk of existential threat, and the brain changes foraging strategies to meet minimal needs in the face of such increased risk (Matisz and Gruber, 2022). This theory predicts that animals with prolonged inflammation would show increased avoidance of potentially threatening contexts. This includes intrinsically avoided contexts, such as open field, and avoidance acquired from association with aversive stimuli (e.g., foot shock). We previously showed that chronic, but not acute, gut inflammation evoked generalization of contextual fear in mice (Matisz et al., 2022). Here, we test for inflammation-evoked potentiation of place avoidance.

Previous studies have demonstrated that somatic inflammation evoked by lipopolysaccharide (LPS) injection reduces fear extinction (Doenni et al., 2017; Pawlik et al., 2023). This was associated with enhanced neural processing of interoceptive fear extinction in regions associated with the fear extinction network, including the striatum. The effects of gut inflammation on aversive learning have received less attention. We are unaware of studies examining the effects of acute or chronic gut inflammation on aversion and extinction. However, the gut microbiome, which is altered in animal models of colitis (Kim et al., 2024; Xu et al., 2021) and human IBD (Abdel-Rahman and Morgan, 2023; Zheng et al., 2024), has been shown to regulate fear extinction learning, and is associated with altered spine remodeling and calcium dynamics in the prefrontal cortex (PFC) (Chu et al., 2019). The involvement of medial PFC (mPFC) and nucleus accumbens (NAc) reported in these studies are consistent with a wealth of data implicating a central role of these neural structures in the contextual control of place preference. For example, rodent studies and human functional neuroimaging studies reveal that the mPFC plays an important role in regulating emotional circuitry involved in conditioned place preference (CPP), and the extinction of conditioned fear (Ochsner et al., 2012; Shiba et al., 2016). The work of Zelinski et al. revealed the contribution of the PFC to discriminative fear conditioning and extinction in rats (Zelinski et al., 2010). Further, ample evidence indicates the NAc is involved in regulating aversive responses, including acquisition and extinction of conditioned aversive responses (Dutta et al., 2021; Ramirez et al., 2015; Wendler et al., 2014). These data suggest that mPFC and NAc are regions of interest in inflammation-evoked changes in place avoidance.

The literature suggests that anxiety and depression are associated with a variety of physiological changes in the brain, including oxidative stress and mitochondrial dysfunction. Chronic inflammation is a well-recognized trigger of these processes (Liu et al., 2023; Rezin et al., 2009). Research indicates that certain regions of the brain, particularly the NAc and mPFC, may be more susceptible to neuroinflammation-induced mitochondrial dysfunction, as evidenced by alterations in metabolism in these regions (Gebara et al., 2021; Matisz and Gruber, 2022; Strasser et al., 2019; Wang et al., 2017). The data in the present study suggest that chronic gut inflammation increases markers of inflammation in both the NAc and mPFC, and increases avoidance of open field and reduces extinction of avoidance of a context previously paired with foot-shock.

## 2 Materials and methods

### 2.1 Animals

The study used 21 male C57Bl/6 mice (8 weeks old, Jackson Laboratories, Bar Harbor, ME, USA). Mice were housed in groups of three in standard clear plastic cages in a vivarium with a 12-h light/dark cycle, with free access to standard mouse chow and drinking water. Animals were acclimatized to the facility for 1 week prior to the start of experimentation. All experiments were carried out in accordance with the guidelines of the Canadian Council of Animal Care, and were approved by the Animal Experimental Ethics Committee of the University of Lethbridge. Because no prior data existed on the effects of chronic dextran sodium sulfate (DSS) colitis on these behavioural outcomes and brain transcripts, this study was used as an exploratory first investigation. Since the effects were unknown and keeping with the principle of minimizing animal use, this initial study is limited to male mice.

### 2.2 Colitis induction

For the induction of DSS colitis, 12 mice were exposed to three cycles (3.0%, 2.75%, 2.75% wt/v) of DSS (MW 40kD; Fisher Scientific, Cat# J14489-22) in their drinking water, each lasting five days, with 9 days of recovery (access to regular drinking water) between the first and the second cycle, and 15 days recovery between the second and third cycle (see Fig. 1). Control mice (n = 9) received regular drinking water (vehicle-treated) for the duration of the experiment. Body weight, fecal levels of lipocalin-2 (LCN2), and disease activity were monitored throughout the experiment as previously described (Nyuyki et al., 2018). Briefly, disease activity was based on three measurements: percentage of body weight lost from the start of the cycle and scored on a 0-4 scale based on the percentage of body weight lost (0 = 0%, 1 = < 1 to ≤ 5%, 2 = > 5 to ≤ 10%, 3 = > 10 to ≤ 15%, 4 = > 15%); stool consistency (solid fecal pellets = 0, soft, sticky stool = 2, loose, watery stool = 4); and the presence of fecal blood (0 = no blood, 4 = fecal blood). The total disease score of each animal was determined as the sum of each of these three measurements, where 0 = normal/unaffected and 12 = maximally affected.

**Fig. 1:**
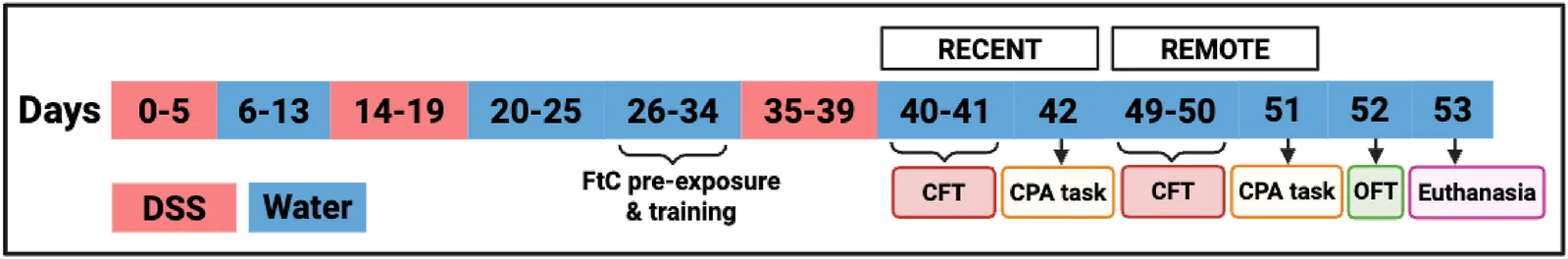
**Experimental Timeline.** Mice were exposed to three cycles of DSS (2.75-3% wt/v), or regular drinking water (vehicle-treated, control mice); CFT, contextual fear training; CPA, conditioned place avoidance; OFT, open field test.

### 2.3 Tissue collection

Following behavioural experiments, mice were euthanized on day 52 by induction into an isoflurane chamber and subsequent injection with ∼1.6 mL/kg Euthanyl (Bimeda MTC 00141704). Colon length and colon and spleen weight were recorded at the time of the necropsy. Brains were immediately removed and snap-frozen via immersion into pre-chilled isopentane for 60-120 s and subsequently stored at −80°C until downstream processing. Tissue punches of two brain regions associated with cognitive, behavioural, and emotional responses, the NAc and the anterior cingulate cortex (ACC), were obtained following protocols outlined by Wager-Miller et al. (2020). Briefly, at least 24 h after removing the brains, the frozen brain was inserted into a frozen brain matrix, and 1 mm coronal slices were made by placing frozen razor blades into the matrix slots. Regions of interest (ROIs) were identified using a mouse brain atlas (Franklin and Paxinos, 2008) and subsequently bilaterally removed by a tissue punch.

### 2.4 RNA isolation and RT-PCR

RNA was extracted using the standard TRIzol™ Reagent protocol (Cat # 1596026, Invitrogen) and reverse transcribed using SuperScript IV™ reverse transcriptase (Cat# 18090010, Invitrogen). The resulting cDNA was analysed by qPCR using 1:10 diluted cDNA with each gene-specific primer. qPCR was performed using the Bio-Rad CFX384 Real-time detection system, with the following thermocycler conditions: 3 min at 95°C (15 s at 95°C, 30 s at 54°C, 30 s at 66°C) × 40 cycles. Fluorometer readings were taken during the extension phase, while standard curves were prepared for relative expression. Quantitative measurements of target gene expression relative to control genes followed the 2^−ΔΔCt^ method. Group differences were expressed as fold changes, normalized to the housekeeping gene hypoxanthine phosphoribosyltransferase (*Hprt*). Gene-specific primers were ordered from IDT (USA, CO) as custom DNA oligomers. The sequences of primers used are shown in Table 1.

**Table 1:** List of RNA primers used in study.

Gene	Forward primer (5′-3′)	Reverse primer (5′-3′)
*Mt-Co1*	TCG CAA TTC CTA CCG GTG TC	CGT GTA GGG TTG CAA GTC AGC
*Gpx1*	CCT CAA GTA CGT CCG ACC TG	CAA TGT CGT TGC GGC ACA CC
*Nrf2*	ACA TTC CCA TTT GTA GAT GAC C	GGT ATT AAG ACA CTG TAA TTC GGG
*Il1b*	TGG TGT GTG ACG TTC CCAT T	CAG CAC GAG GCT TTT TTG TTG
*Hprt*	TCC TCC TCA GAC CGC TTT T	CCT GGT TCA TCA TCG CTA ATC

### 2.5 ELISA

Fecal LCN2 is a biomarker for intestinal inflammation in humans and rodents (Chassaing et al., 2012). Samples were prepared following previously published protocols (Matisz et al., 2022). Briefly, frozen fecal samples were reconstituted in 1% Tween 20 in PBS, centrifuged, and the supernatants were collected and stored at −20°C until analysis. LCN2 levels were quantified in the supernatants using the DuoSet murine LCN2 ELISA Kit (R&D Systems, Minneapolis, MN), following the manufacturer's instructions.

### 2.6 Behavioural tests

#### 2.6.1 Contextual fear discrimination & conditioned place avoidance tasks

Contextual fear discrimination was used to assess associative memory by measuring freezing behaviour in a shock-paired context versus a novel unpaired context distinguished by different spatial and visual cues. The experimental design follows Zelinski et al. (2010) and Matisz et al. (2022), with minor modifications. *Apparatus:* A rectangular chamber (30.3 cm × 26.2 cm x 30.5 cm) with solid-white walls, and an equilateral triangular shaped chamber (26.3 cm × 30.5 cm) with black and white-striped walls were constructed from Plexiglas, producing contexts with different spatial and visual cues. Amylacetate (Sigma-Aldrich, #W504009) and VapoRub™ (Shopper's Drug Mart, Lethbridge, Canada) were used as the olfactory cues for the square and triangle contexts, respectively. Both chambers included a small door, where a removable Plexiglas hallway connected the two chambers. The flooring of the chambers and the alleyway consisted of parallel metal rods (0.2 cm diameter) placed 0.4 cm apart. The entire apparatus was placed on a clear Plexiglas table, 21 cm from the floor, with an ELP 2.0 MP 5-50 mm USB camera (Shenzhen Ailipu technology Co., Ltd, Shenzhen, Guangdong, China) placed underneath the table to record animal movements.

*Pre-exposure:* Prior to training, animals were pre-exposed to the apparatus to reveal any chamber biases. Animals were placed in the centre of the Plexiglas hallway that connected both chambers and allowed to freely explore for 10 min. The dwell time in each chamber was recorded and summated by an observer. The preferred chamber, as indicated by dwell time greater than 50%, was paired with a mild foot shock.

*Training:* Experimental training began approximately 24 h after pre-exposure to the apparatus. During training, Plexiglas panels were inserted to block access to the connecting hallway. Each animal was placed in one of the chambers for 5 min. In the paired context, mice received a 2 s 0.5 mA foot shock delivered at minute 2, 3, and 4. No foot shock was delivered in the unpaired context. Paired and unpaired contexts were located in separate rooms. Animals were exposed to the paired or unpaired context in an alternating fashion for a total of 8 days. The training schedule (order of exposure to context), and the chamber paired with foot shock were counterbalanced within and between treatment groups to the best of our ability.

*Testing:* The testing procedure took place over two days in the room where unpaired training occurred at a recent (day 40-41) and remote (day 49-50) timepoint. Animals were placed in their assigned context and video recorded for 5 min. On the subsequent day, animals were placed in the alternate context, and video recorded for 5 min. No foot shocks were delivered during either testing session. Freeze-time scoring for contextual fear behavioural tasks was scored using a combination of DeepLabCut (Mathis et al., 2018; Lauer et al., 2022) and a custom MATLAB script; these values were validated by an experienced observer blind to treatment groups.

#### 2.6.2 Conditioned place avoidance

As a measure of active avoidance behaviour and aversive memory expression, on days 42 and 51, approximately 24 h after conclusion of the contextual fear training (CFT), animal contextual aversion was assessed in the same apparatus and room where CFT occurred. Mice were placed in the centre of the Plexiglas hallway, which connected both the paired and unpaired context chambers. They were allowed to roam freely between the contexts for 10 min while being recorded with a floor-mounted camera (ELP 2.0 MP 5-50 mm USB). The total time spent in each context was recorded by an experienced observer blind to experimental conditions.

#### 2.6.3 Open Field Test

Anxiety-like behaviour, novel environment exploration, and general locomotor activity were assessed on day 52 by recording time spent in the centre of an open field and total distance travelled. Mice were placed in the centre of the open field arena (50L x 50W × 25H cm; constructed from white Plexiglas) and allowed to roam freely for 10 min. Animal behaviour was recorded with a ceiling-mounted ELP 2.0 MP 5-50 mm USB camera. The open field test (OFT) was scored using a custom tracking algorithm. The tracking algorithm used OpenCV's implementation of a mixture of Gaussians 2 algorithm (MOG2). The algorithm tracked the centre of mass of the animal as it moved through the arena. Position was used to compute centre dwell time (innermost 25 × 25 cm defined as the centre) and total distance travelled. Anxiety-related behaviour was scored as time spent in the centre relative to vehicle treated mice, and locomotion was represented as the total distance travelled.

### 2.7 Statistical analysis

All analyses were performed in GraphPad Prism 10.0.1, graphs were created in the same software. Data are presented as mean ± SEM. Group sizes were n = 9 vehicle treated and n = 12 DSS-treated male mice, except colon length (n = 9 and n = 11) and brain gene expression (n = 6 per group, with paired ACC and NAc samples from each animal). Body weight across the 55-day time course was analysed via two-way repeated measures ANOVA with Tukey's multiple-comparisons test. Colon length, and colon- and spleen-to-body weight ratios were compared between vehicle- and DSS-treated mice using unpaired t-tests.

To accommodate for a small number of missing values in longitudinal fecal sampling, LCN2 concentrations were analysed via mixed effects analysis with Sidak's multiple-comparisons test.

Behavioural data were analysed by two-way repeated measures ANOVA. Dwell time in the paired and unpaired contexts at the recent and remote time points was followed by Tukey's multiple-comparisons test, as was the proportion of time spent in the paired context across time points. Distance travelled in the paired and unpaired contexts was followed by Sidak's multiple-comparisons test. Reduction in freezing across time points within each treatment group was assessed by one-sample *t*-test. Time spent in the centre and total distance travelled in the OFT was compared by unpaired *t*-test. The relationship between fecal LCN2 and OFT centre time in DSS-treated mice was evaluated through simple linear regression.

Changes in brain gene mRNA expression (*Nrf2, Mt-Co1, Il1b, Gpx1*) were analysed by two-way repeated measures ANOVA followed by Sidak's multiple comparisons test.

## 3 Results

### 3.1 Cyclic exposure to DSS produced signs of gut inflammation and disease

The addition of DSS to drinking water of mice reliably evokes inflammation of the colon and is a widely used model of ulcerative colitis with excellent face validity (Chassaing et al., 2014). Although many studies have utilized an acute DSS paradigm consisting of a single 5–7-day treatment, multiple chronic DSS protocols have also been established (Okayasu et al., 1990; Cooper et al., 1993; Clapper et al., 2007; Wirtz et al., 2017). In the present study, we employed a chronic DSS paradigm of three DSS cycles over a 40-day period. As expected, mice exposed to DSS had significantly lower body weight relative to vehicle-treated mice (Fig. 2A main effect of time, F_2.35,44.69_ = 9.90, p < 0.001 main effect of treatment, F_1,19_ = 12.99, p = 0.002; interaction, F_2.35,44.69_ = 7.08, p = 0.001). Levels of fecal LCN2, a marker of gut inflammation, were significantly elevated in DSS-treated mice throughout the study (Fig. 2B main effect of time, F_2.2, 51.12_ = 19.56; main effect of treatment, F_1,93_ = 86.19; interaction, F_2.2, 51.12_ = 19.56; all p < 0.001). DSS-induced intestinal epithelial injury results in reproducible shortening of the colon due to thinning of the intestinal wall, immune cell infiltration, and fibrosis that causes the colon to contract (Chassaing et al., 2014). Additionally, spleen weight acts as a marker for systemic inflammatory burden, since inflammation leads to expansion and activation of immune cell populations (Nunes et al., 2018). As expected, colon length was significantly shorter in mice with DSS (Fig. 2C; t = 4.44, df = 18, p < 0.001). When normalized to body weight, their colon weight (t = 3.74, df = 19; p = 0.001) and spleen weight (t = 3.19, df = 19; p = 0.005) were significantly elevated relative to vehicle-treated mice (Fig. 2D and E). These are all hallmarks of significant gut inflammation (Chassaing et al., 2014).

**Fig. 2:**
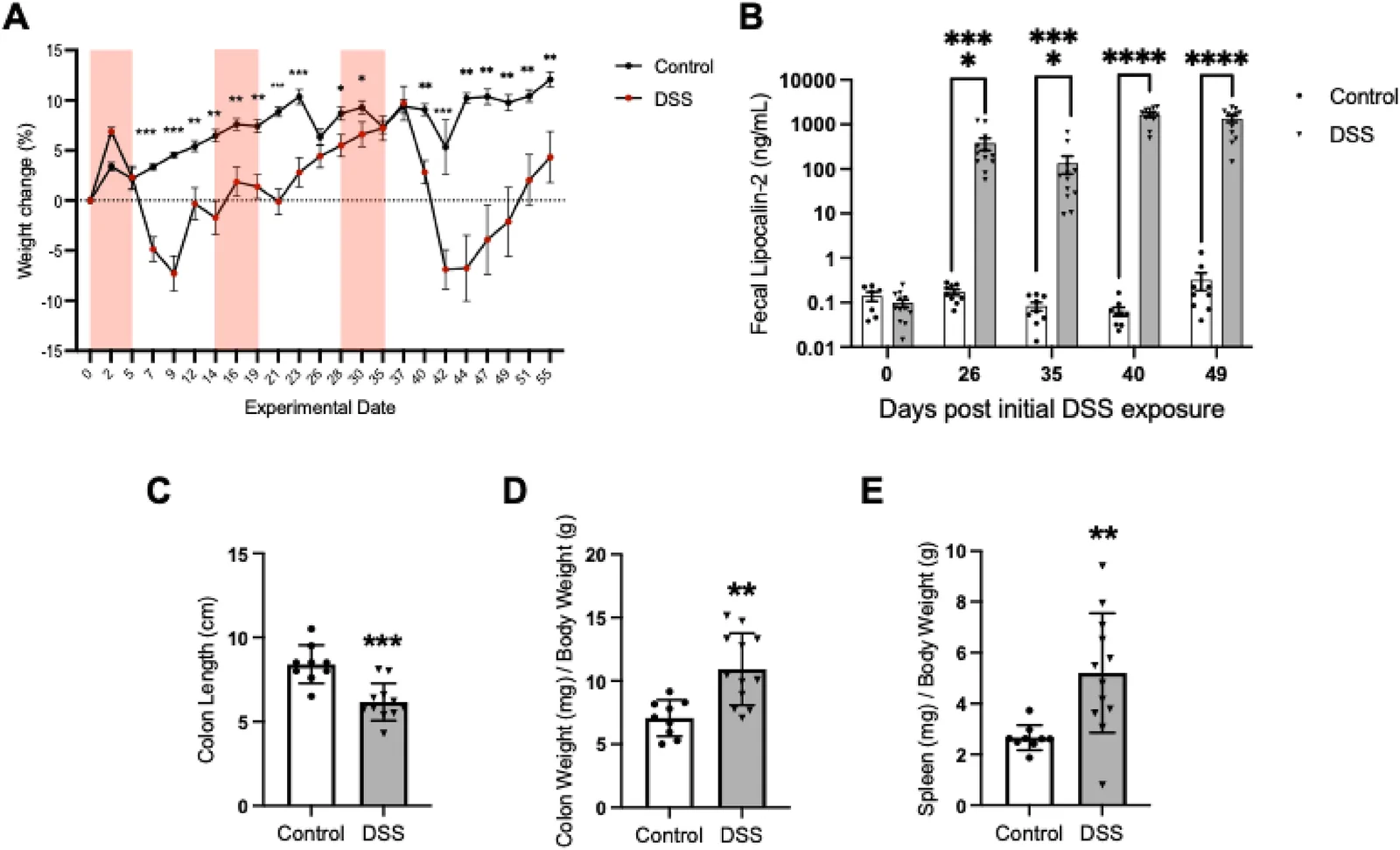
**Exposure to DSS produces typical signs of gut inflammation.** (A) Percent change in body weight throughout the duration of the experiment. (B) Fecal LCN2 expression throughout the study. (C) Colon length at time of necropsy (day 52). (D) Ratio of colon and (E) spleen to body weight. Data are mean ± SEM; n = 9 control and n = 12 DSS male mice per treatment group. *p < 0.05; **p < 0.01; ***p < 0.001; ****p < 0.0001.

### 3.2 Mice with DSS-colitis exhibited prolonged aversion to contexts associated with foot shock

Mice underwent associative training in two testing chambers with distinct contextual cues. One chamber was paired with mild foot shock, whereas the other was unpaired. In subsequent testing, mice had free access to both contexts so that we could assess aversion by asymmetry in dwell time among chambers. Both vehicle-treated and DSS-treated mice spent less time in the shock-paired context than the unpaired context at both the recent (Fig. 3A; F_1,19_ = 301.1; vehicle-treated p < 0.001; DSS p < 0.001) and remote (Fig. 3B; F_1,19_ = 66.52; vehicle-treated p = 0.001; DSS p < 0.001) time points. Interestingly, vehicle-treated mice appeared to show extinction of aversion whereas DSS-treated mice did not. Vehicle-treated mice significantly increased the amount of time they spent in the paired context during the remote time point as compared to the recent time point 7 days prior (Fig. 3C main effect of time, F_1,18_ = 19, p < 0.001; interaction, F_1,18_ = 13.44, p = 0.002. Tukey post-test: recent vs remote for vehicle-treated mice, p < 0.001). In contrast, DSS-treated mice did not increase dwell time in the paired context during the later test (p = 0.61). Both groups spent similar proportions of time in the paired context at the recent timepoint (p = 0.28), whereas vehicle-treated mice spent significantly more time in the paired context at the remote timepoint (p = 0.001). Overall, these results suggest that vehicle-treated mice extinguish their aversion to the shock-associated chamber, but the DSS-treated mice do not. This is further supported by analysis of freezing when mice are confined in each of the two contexts. The vehicle-treated mice exhibited reduced freezing in the unpaired context upon the second test one week after the first (one-sample *t*-test, t = 2.32, p = 0.049). In contrast, DSS-treated mice did not reduce freezing in the latter testing (one sample *t*-test, t = 0.04, p = 0.97).

**Fig. 3:**
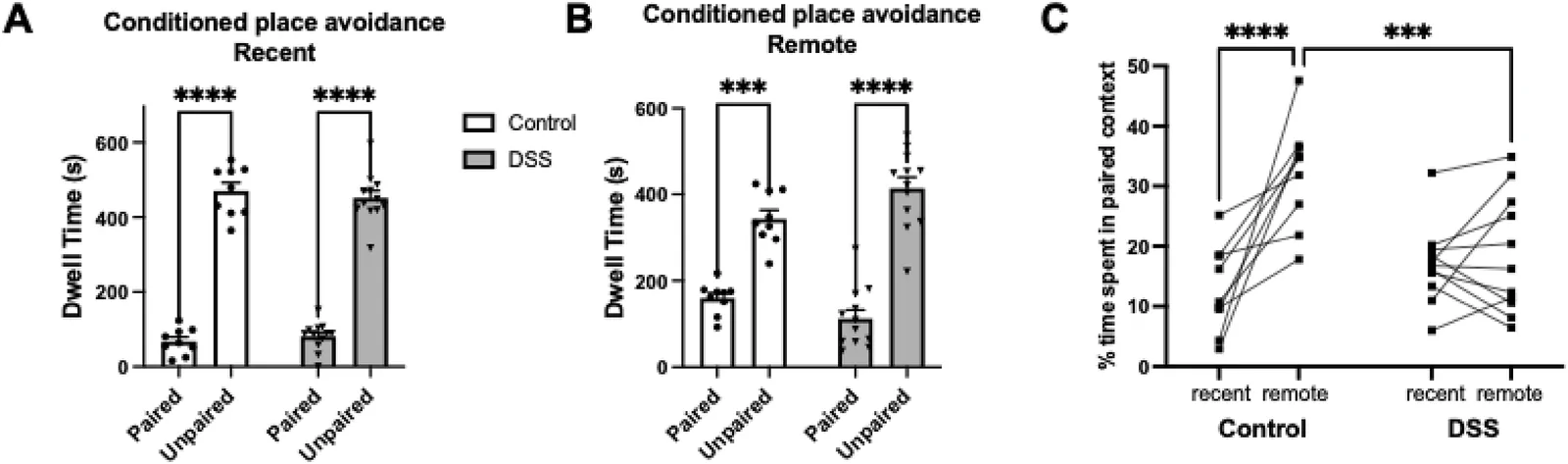
**Context preference at recent and remote timepoints.** Amount of time spent in the paired and unpaired contexts at the (A) recent, and (B) remote time points. (C) Proportion of time spent in paired context at recent and remote time points. Data are mean ± SEM; n = 9 control and n = 12 DSS male mice per treatment group. *p < 0.05; **p < 0.01; ***p < 0.001; ****p < 0.0001.

### 3.3 DSS-treated mice exhibited reduced locomotion in both conditioned contexts

We next assessed the distance travelled in paired and unpaired contexts at recent (Fig. 4A) and remote (Fig. 4B) time points. At the recent timepoint, we observed a strong effect of context, particularly for the DSS-treated animals (main effect of treatment, F_1,19_ = 0.21, p = 0.65; main effect of context, F_1,19_ = 5.17, p = 0.035; interaction, F_1,19_ = 3.09, p = 0.095). The vehicle-treated mice travelled equal distances in the paired and unpaired contexts (Sidak's post-hoc test, p = 0.74); conversely, DSS mice travelled significantly more in the unpaired context (Sidak's post-hoc test, p = 0.006). When behaviours were tested again 7 days later, locomotion varied significantly by treatment group and context (main effect of treatment, p = 0.002; main effect of context, p < 0.001; interaction, p = 0.49). Both treatment groups travelled greater distances in the unpaired context relative to the paired context (Sidak's post hoc test, vehicle-treated, p = 0.033; DSS, p = 0.001), with vehicle-treated mice travelling significantly more than DSS mice in both their paired (Sidak's post hoc test, p = 0.002) and unpaired (Sidak's post hoc test, p = 0.012) contexts. Additionally, freezing times differed in paired and unpaired contexts during recent and remote time points (see Supplementary Fig. 2).

**Fig. 4:**
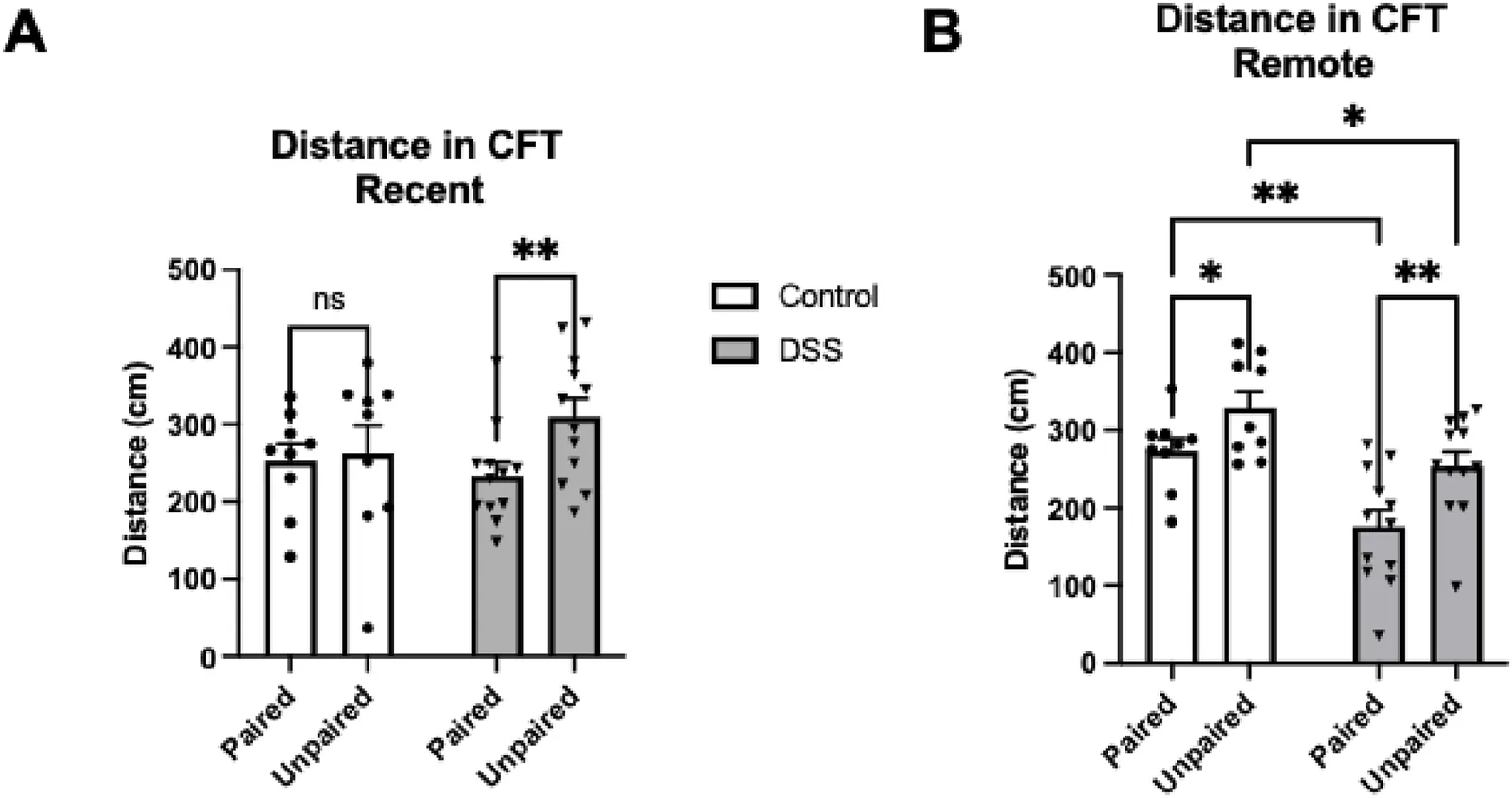
**Distance travelled in paired and unpaired contexts.** Total distance travelled in paired and unpaired contexts at the (A) recent and (B) remote time point. Data are mean ± SEM; n = 9 control and n = 12 DSS male mice per treatment group. *p < 0.05; **p < 0.01. CFT, contextual fear training.

### 3.4 DSS-treated mice avoided the centre of an open field, a classic endophenotype of anxiety

Approximately 24 h following conditioned place avoidance (CPA) testing at the remote time point, mice were tested in an open field apparatus. DSS-treated mice displayed increased aversion to the centre of the field, relative to vehicle-treated mice (Fig. 5A; t = 2.28, df = 19, p = 0.034). To test if this was a consequence of reduced locomotion, we compared the distance travelled in the open field. Both treatment groups travelled similar distances (Fig. 5B; t = 0.19, df = 19, p = 0.85). Among DSS-treated mice, the time spent in the centre of the arena was significantly anti-correlated with fecal LCN2 levels (Fig. 5C; r^2^ = 0.44, p = 0.018); no such relationship existed among controls (Supplementary Fig. 1; r^2^ = 0.19, p = 0.24). This suggests that animals with more severe gut inflammation were more averse to the centre of the field. In sum, the DSS-treated mice exhibited locomotion comparable to vehicle-treated mice in the OFT. This contrasts with the reduced locomotion by these mice in the CPA test.

**Fig. 5:**
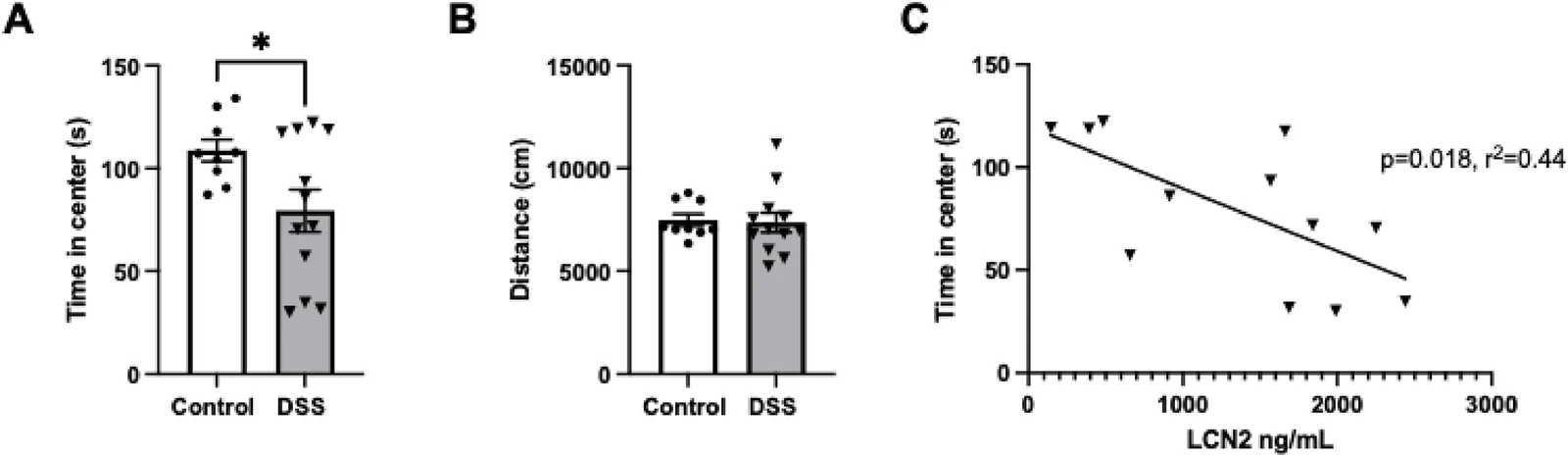
**Behaviour in the Open Field Test.** (A) Time spent in centre. (B) Distance travelled. (C) Correlation between time spent in the centre and fecal LCN2 among animals exposed to DSS. Data are mean ± SEM; n = 9 control and n = 12 DSS male mice per treatment group. *p < 0.05.

### 3.5 DSS treatment was associated with gene transcript changes in the medial prefrontal cortex and ventral striatum

We used RT-PCR to quantify transcripts of several genes of interest in the NAc of the ventral striatum, and ACC region of mPFC. We chose to assess the transcript levels of a set of complementary genes. The proinflammatory cytokine interleukin-1beta (IL-1B) protein is a key mediator of the neuroinflammatory response in the brain. We use the expression of the IL-1B gene (*Il1b*) as an indicator of region-specific inflammation in the DSS-treated mice. Mitochondrial DNA (mtDNA) encodes the cytochrome-c oxidase subunit 1 (*Mt-Co1*) gene, a critical component of the electron transport chain required for oxidative phosphorylation and cellular energy production (Brischigliaro and Zeviani, 2021). Inflammation can promote oxidative stress, which must be tightly regulated to limit cell damage and pathology. Therefore, we selected two transcripts to assess oxidative stress responses in the brain. Nuclear factor erythroid 2 related factor 2 (*Nrf2*) regulates antioxidant response elements (Ngo and Duennwald, 2022), and *Gpx1* encodes for glutathione peroxidase 1, an enzyme that works with glutathione as a key neuronal antioxidant (Lubos et al., 2011).

DSS treatment was associated with significantly higher expression of *Nrf2* in the ACC (p = 0.041), but not the NAc (p = 0.32; Fig. 6A; main effect of structure, F_1,10_ = 7.6, p = 0.02; main effect of treatment, F_1,10_ = 5.0, p = 0.049). Expression of *Il1b* was significantly higher among DSS-treated animals in the NAc (p = 0.007), but not the ACC (p = 0.59; Fig. 6C; main effect of structure, F_1,10_ = 9.14, p = 0.013; main effect of structure and treatment interaction, F_1,10_ = 7.34, p = 0.022). In the present study, DSS treatment did not affect expression of *Mt-Co1,* or *Gpx1* in either region. In addition to the structure-specific effects of DSS described above, we also observed expression differences among structures in vehicle-treated animals (see Supplementary Fig. 3). Notably, the NAc had significantly greater expression of *Nrf2* (t = 2.55, p = 0.029), *Mt-Co1* (t = 5.72, p < 0.001), and *Gpx1* (t = 4.44, p = 0.001) compared to the ACC. Conversely, the expression of *Il1b* (t = 4.01, p = 0.003) was significantly lower in the NAc than ACC. The functional impacts of these differences are not well understood.

**Fig. 6:**
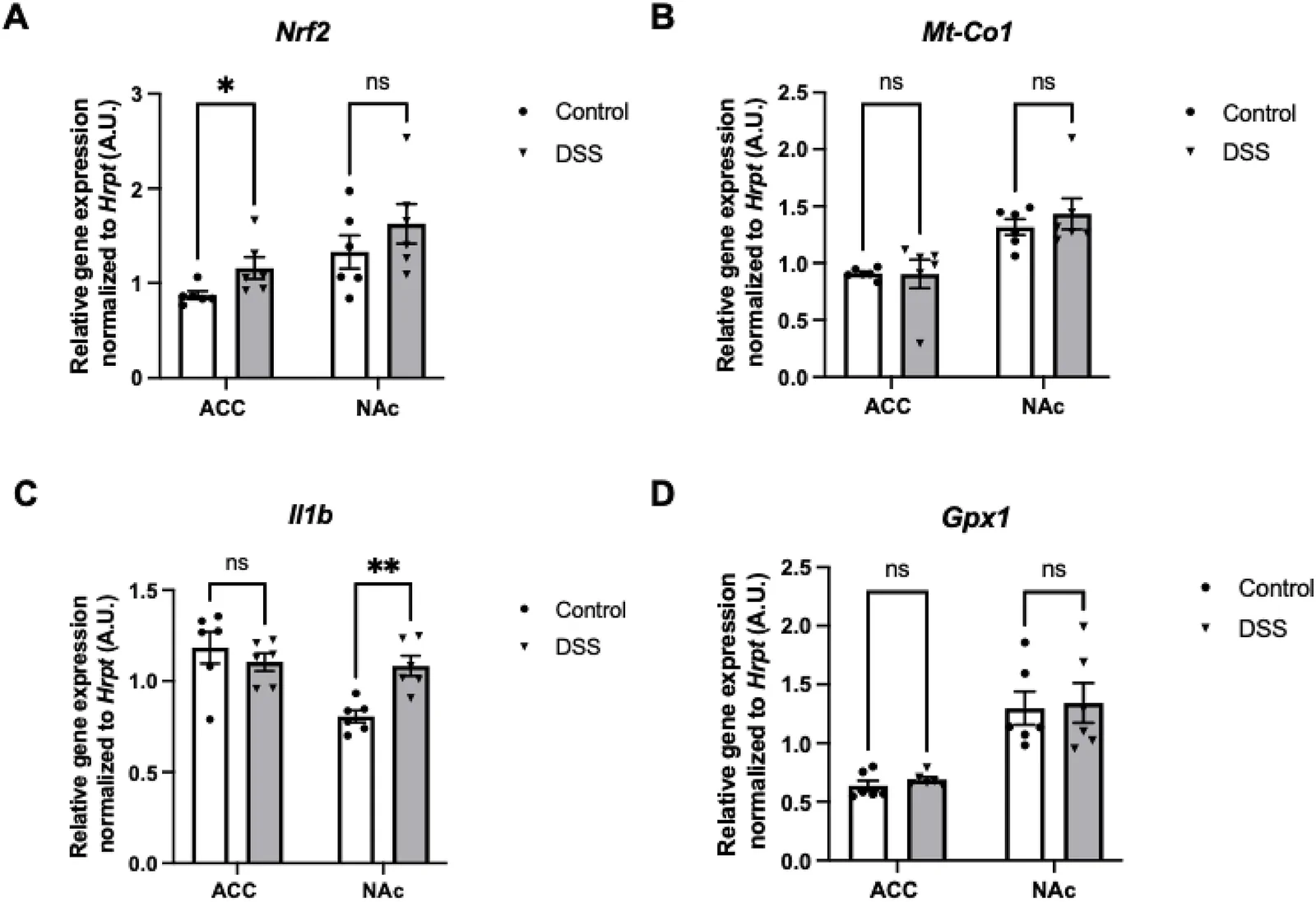
**Expression of mRNA transcripts in the ACC and NAc in control and DSS mice.** (A) Mean expression of (A) *Nrf2* (B) *Mt-Co1* (C) *Il1b* and (D) *Gpx1*, using *Hprt* as a reference gene, in the ACC and NAc. Data represent mean ± SEM; n = 6 per treatment group. *p < 0.05, **p < 0.01.

## 4 Discussion

If illness and injury raise an animal's exposure to threat, then the brain should respond by shifting behaviour towards risk aversion by narrowing exploration, sustaining avoidance of contexts that signal danger, and meeting minimal needs rather than maximizing gain (Hart, 1988; Matisz et al., 2022; Matisz and Gruber, 2022). This model makes a specific and testable prediction: chronic inflammation should potentiate avoidance of threatening contexts whether that threat is learned or innate. In this study we investigated the effects of chronic gut inflammation on intrinsic aversion to open field and on aversion to a context-specific chamber associated with foot shock, an assay used to model anxiety-like behaviours (Zelinski et al., 2010). The present data are consistent with that prediction on both counts. Chronic gut inflammation increased avoidance of the centre of an open field and slowed the decline of avoidance of a context previously paired with foot shock when tested at a later timepoint. These behavioural effects were accompanied by brain region-specific changes in transcripts associated with inflammatory and antioxidant responses in two structures implicated in contextual control of behaviour. We extend our previous work in this model, which established that chronic gut inflammation generalizes contextual fear (Matisz et al., 2022) and reshapes transcript expression across limbic structures (Matisz et al., 2025), by showing that the behavioural consequence persists at a remote timepoint, extends to an unlearned aversive context, and scales with the severity of peripheral inflammation.

The parallel findings of the two behavioural assays are the central result. Avoidance of the open-field centre requires no training and reflects an unlearned assessment of risk, whereas avoidance of the shock-paired chamber is acquired and context-specific. Chronic gut inflammation potentiated both. Critically, these findings are not independent of disease severity: among DSS-treated animals, the time spent in the centre of the open field was inversely related to fecal LCN2, an established index of intestinal inflammation (Chassaing et al., 2012). Animals with more severe colitis were more avoidant. Increased centre avoidance has been reported in acute colitis and other models of intestinal inflammation (Dempsey et al., 2019; Hassan et al., 2014; Holder et al., 2019; Lyte et al., 2006; Nyuyki et al., 2018; Wei et al., 2019; Xu et al., 2018). These studies underscore how gut inflammation can produce anxiety-like phenotypes, an association that is well reported in clinical literature (Bannaga and Selinger, 2015; Matisz and Gruber, 2022). What is added here is that the effect persists under a chronic, cyclical inflammatory course and tracks with severity of peripheral inflammation.

This potentiated avoidance is unlikely to reflect generalized sickness-related hypoactivity. Locomotion in the CPA apparatus was indistinguishable between treatment groups at the recent timepoint and lower among DSS-treated mice at the remote timepoint in both the paired and unpaired chambers. Yet when the animals were placed in the OFT apparatus the following day, DSS-treated and vehicle-treated mice travelled similar distances. A global motor deficit therefore cannot produce the potentiated avoidance observed in DSS-treated mice during the CPA task. Instead, this pattern may suggest that reduced locomotion is context-dependent, and two explanations are compatible with our data. Firstly, fear generalized to the unpaired chamber in this model previously (Matisz et al., 2022), which would make both contexts threat-associated and suppress movement in each. Alternatively, mice with gut inflammation may adjust exploratory strategy according to the context novelty. Indeed, previous studies have demonstrated that animals increase locomotion in novel environments (Lenow et al., 2017), and the open field in the present study was novel whereas the conditioning chambers had been encountered repeatedly over the study. To our knowledge, the effects of chronic gut inflammation on locomotion have not been tested before. We speculate that context-dependent locomotor behaviours that emerge in mice with chronic gut inflammation may be a consequence of changes to neuronal circuits involved in motivation, or affect, although further work is required to test this hypothesis (Felger and Treadway, 2017; Nyuyki et al., 2018; Matisz et al., 2022; Wu et al., 2023). However, centre-avoidance effect arose in the one setting where locomotion was unaffected, which is why we interpret these findings as a change in threat appraisal rather than in motor output.

What chronic gut inflammation did not alter is as informative as what it did. Both treatment groups preferred the unpaired from the paired context during the CPA task at both timepoints, indicating that learning the association and its context were intact. However, the groups diverged in how that context avoidance changed between the recent to the remote timepoint. Compared to the recent time point, vehicle-treated mice spent significantly more time in the paired context at the remote time point, whereas DSS-treated mice did not and exhibited similar rates of aversion to the paired context between the recent and later time point. Notably, responses among DSS-treated mice were heterogeneous at the remote timepoint with some animals increasing their time in the paired context comparable to the vehicle-treated mice, and others showing no change. Because centre avoidance in the open field scaled with LCN2 in these same animals, individual differences in inflammatory severity are a candidate mechanism for this variability. Chronic gut inflammation therefore appears to impair the fear extinction process rather than the acquisition process. This interpretation is consistent with the limited previous work on this topic. For example, acute inflammation impaired the extinction of a visceral pain-related fear memory in healthy humans in a double-blind, placebo-controlled study (Benson et al., 2020), and inflammation reshapes neural processing during interoceptive fear extinction in regions including the striatum (Pawlik et al., 2023). We are not aware of a comparable demonstration under chronic gut inflammation.

Against the behavioural background, the transcript data indicate that peripheral inflammation does not act uniformly on the brain but produces distinct regional signatures. The NAc and ACC differ at baseline in our vehicle-treated mice, with higher *Mt-Co1*, *Nrf2*, and *Gpx1* in the NAc, replicating our earlier observations (Matisz et al., 2025) and consistent with the broader heterogeneity of the brain in cell composition, connectivity, and bioenergetic demand (Anderson et al., 2013; Grabert et al., 2016; Ochocka and Kaminska, 2021; Rosenberg et al., 2023). DSS treatment had two non-overlapping effects: elevated *Il1b* in the NAc and elevated *Nrf2* in the ACC. The NAc effect is expected from the microglial route by which peripheral signals are translated into central inflammatory tone (Liddelow et al., 2017; Süß et al., 2020). Furthermore, colitis has been reported to raise *Il1b* in the hippocampus (Gadotti et al., 2019; Zonis et al., 2015) and lateral habenula (Hu et al., 2020; Zheng et al., 2023), alter inflammatory gene expression in isolated microglia (Caetano-Silva et al., 2024), and similar effects have been reported in other inflammatory models (Talley et al., 2021; Zhao et al., 2019). Our findings extend this observation to the ventral striatum.

The effect of DSS treatment on the ACC is of a different character. *Nrf2* is an important regulator of antioxidant responses and mitochondrial function in the brain (Bell et al., 2015; Esteras and Abramov, 2022; Khor et al., 2006; Ngo and Duennwald, 2022; Sandberg et al., 2014). Here, its elevation without an accompanying change in *Gpx1* or *Mt-Co1* is tentatively interpreted as an engaged and compensatory redox response rather than as evidence of established oxidative damage and mitochondrial failure. The ACC showing this response in the present study is consistent with clinical evidence that the ACC is disproportionately affected in IBD and irritable bowel syndrome (Matisz and Gruber, 2022) and with our observation that transcript expression in this region tracks peripheral inflammatory severity more closely than in other regions sampled (Matisz et al., 2025).

Linking these molecular signatures to behaviour is where our data are most limited, and we advance the following only as a hypothesis rather than a conclusion. The NAc mediates the acquisition and extinction of conditioned aversive stimuli (Dutta et al., 2021; Ramirez et al., 2015; Wendler et al., 2014) and with the ACC, supports context-dependent cognitive, behavioural, and emotional responses (Gruber and McDonald, 2012; Mancini et al., 2021). Elevated *Il1b* mRNA in the NAc therefore puts an inflammatory signal in the circuitry that the observed CPA behavioural phenotype implicates: DSS-treated mice acquired and discriminated the aversive context but failed to update that association over time. We attribute this to a deficit in fear extinction rather than learning. We did not test for a relationship between *Il1b* expression and avoidance behaviour, and the present data cannot establish whether this transcriptional change contributes to the behavioural change or is unrelated to it. Regionally restricted interference with IL-1B signalling in the NAc during the extinction interval would distinguish these possibilities.

The duration of intestinal inflammation is important when comparing these findings with prior work. Affective changes have been reported during and after a single DSS course, including anxiety-like behavior and persistent central inflammatory responses during recovery (Dempsey et al., 2019) and conditioned preference for relief of colitis-associated discomfort (Chen et al., 2015). In our earlier three-cycle DSS study, mice were tested for shock-context preference at recent and remote intervals; chronic DSS increased remote fear generalization while preference for the unpaired context remained intact (Matisz et al., 2022). Region-dependent brain transcriptional changes have likewise been reported after repeated DSS cycles in a separate cohort (Matisz et al., 2025). The present cohort extends this work by examining learned and unconditioned avoidance alongside serial indices of gut inflammation and terminal, targeted ACC and NAc transcript measurements. The treatment-by-time difference in place avoidance and the relation between open-field centre time and fecal LCN2 support a broader effect of chronic DSS on avoidance behavior. The absence of an acute DSS group limits inference about whether these effects are specific to prolonged inflammation.

Several limitations constrain these findings. The study design is correlational, and no manipulation tested necessity or sufficiency. Circulating markers of inflammation were not assayed, so the route by which peripheral inflammation reached the brain cannot be specified. Our molecular measurements are of transcript abundance, and we did not test protein levels, oxidative stress products, or assess mitochondrial function. Changes in transcript levels do not necessarily translate to changes in the corresponding proteins, or in the processes that they regulate. The transcriptional changes are modest in magnitude and measured in whole-tissue punches and no cell-type isolation was performed, so they represent a regional shift in expression rather than a demonstrated change in physiological function or specific cell type change in expression. The interpretations of altered transcript levels in the ACC and NAc remain provisional, and future work requires local manipulation of NRF2 and IL-1B signalling to characterize their relationships with potentiated intrinsic and context avoidance in a model of chronic gut inflammation. Furthermore, since only male mice were used in the present study, generalization to females remains untested.

## 5 Conclusions

In summary, the present data suggest that chronic inflammation of the gut reduced extinction of place avoidance and potentiated intrinsic avoidance, effects that are not explained by changes in locomotor output. Further, DSS-colitis evoked heterogeneous changes of gene transcription among limbic brain structures. Our results are consistent with the possibility that chronic peripheral inflammation is accompanied by region-specific transcriptional changes in the brain that co-occur with increased avoidance behaviour and underscore the importance of understanding the biological processes by which peripheral inflammation can lead to anxiety and mood disorders among people with IBD.

## CRediT authorship contribution statement

**B. Cej:** Writing – review & editing, Formal analysis, Data curation, Visualization. **K. Beekman:** Writing – original draft, Methodology, Investigation, Formal analysis, Data curation, Conceptualization. **C.E. Matisz:** Writing – original draft, Visualization, Validation, Supervision, Resources, Project administration, Methodology, Investigation, Funding acquisition, Formal analysis, Data curation, Conceptualization. **T. Haight:** Resources, Methodology, Data curation. **A. Zovoilis:** Writing – review & editing, Resources. **R.J. McDonald:** Writing – review & editing, Resources. **A.J. Gruber:** Writing – review & editing, Validation, Supervision, Software, Resources, Project administration, Methodology, Investigation, Funding acquisition, Formal analysis, Data curation, Conceptualization.

## Ethics declaration

This study was conducted in accordance with the following guidelines for animal welfare and/or reporting: Canadian Council on Animal Care. This study was approved by the Animal Experimental Ethics Committee of the University of Lethbridge.

## Ethics approval

All experiments were carried out in accordance with the guidelines of the Canadian Council of Animal Care and were approved by the University of Lethbridge Animal Care Committee (Protocol #2018).

## Funding

AJG received an NSERC Discovery Grant from the 10.13039/501100000038Natural Sciences and Engineering Research Council of Canada. AJG is also supported by the Alberta Children’s Research Institute, Azrieli Foundation, and Hotchkiss Brain Institute. AZ is a recipient of the Canada Research Chairs program and Genome Canada BioNet grant. CEM was supported by an 10.13039/501100000038NSERC PDF.

## Declaration of competing interest

The authors declare that they have no known competing financial interests or personal relationships that could have appeared to influence the work reported in this paper.

## Data Availability

All data supporting described findings can be obtained from the corresponding author (AJG) upon reasonable request.

## References

[bib1] Abdel-Rahman L.I.H., Morgan X.C. (2023). Searching for a consensus among inflammatory bowel disease studies: a systematic meta-analysis. Inflamm. Bowel Dis..

[bib2] Anderson M.L., Kinnison J., Pessoa L. (2013). Describing functional diversity of brain regions and brain networks. Neuroimage.

[bib3] Bannaga A., Selinger C.P. (2015). Inflammatory bowel disease and anxiety: links, risks, and challenges faced. Clin. Exp. Gastroenterol..

[bib4] Barberio B., Zamani M., Black C.J., Savarino E.V., Ford A.C. (2021). Prevalence of symptoms of anxiety and depression in patients with inflammatory bowel disease: a systematic review and meta-analysis. Lancet Gastroenterol. Hepatol..

[bib5] Bell K.F.S., Al-Mubarak B., Martel M.-A., McKay S., Wheelan N., Hasel P., Márkus N.M., Baxter P., Deighton R.F., Serio A., Bilican B., Chowdhry S., Meakin P.J., Ashford M.L.J., Wyllie D.J.A., Scannevin R.H., Chandran S., Hayes J.D., Hardingham G.E. (2015). Neuronal development is promoted by weakened intrinsic antioxidant defences due to epigenetic repression of Nrf2. Nat. Commun..

[bib6] Benson S., Rebernik L., Pastoors D., Brinkhoff A., Wegner A., Elsenbruch S., Engler H. (2020). Impact of acute inflammation on the extinction of aversive gut memories. Brain Behav. Immun..

[bib7] Brischigliaro M., Zeviani M. (2021). Cytochrome c oxidase deficiency. Biochimica et Biophysica Acta (BBA) - Bioenergetics.

[bib8] Caetano-Silva M.E., Rund L., Vailati-Riboni M., Matt S., Soto-Diaz K., Beever J., Allen J.M., Woods J.A., Steelman A.J., Johnson R.W. (2024). The emergence of inflammatory microglia during gut inflammation is not affected by FFAR2 expression in intestinal epithelial cells or peripheral myeloid cells. Brain Behav. Immun..

[bib9] Chassaing B., Aitken J.D., Malleshappa M., Vijay‐Kumar M. (2014). Dextran sulfate sodium (DSS)‐Induced colitis in mice. Curr. Protoc. Im..

[bib10] Chassaing B., Srinivasan G., Delgado M.A., Young A.N., Gewirtz A.T., Vijay-Kumar M. (2012). Fecal lipocalin 2, a sensitive and broadly dynamic non-invasive biomarker for intestinal inflammation. PLoS One.

[bib75] Chen J., Winston J.H., Fu Y., Guptarak J., Jensen K.L., Shi X.Z., Green T.A., Sarna S.K. (2015). Genesis of anxiety, depression, and ongoing abdominal discomfort in ulcerative colitis-like colon inflammation. Am. J. Physiol. Regul. Integr. Comp. Physiol..

[bib11] Chu C., Murdock M.H., Jing D., Won T.H., Chung H., Kressel A.M., Tsaava T., Addorisio M.E., Putzel G.G., Zhou L., Bessman N.J., Yang R., Moriyama S., Parkhurst C.N., Li A., Meyer H.C., Teng F., Chavan S.S., Tracey K.J. (2019). The microbiota regulate neuronal function and fear extinction learning. Nature.

[bib12] Clapper M.L., Cooper H.S., Chang W.-C.L. (2007). Dextran sulfate sodium-induced colitis-associated neoplasia: a promising model for the development of chemopreventive interventions. Acta Pharmacol. Sin..

[bib13] Cooper H.S., Murthy S.N., Shah R.S., Sedergran D.J. (1993). Clinicopathologic study of dextran sulfate sodium experimental murine colitis. Lab. Investig. J. Techn. Methods Pathol..

[bib16] D'Mello C., Swain M.G., Dantzer R., Capuron L. (2016).

[bib14] Dempsey E., Abautret-Daly Á., Docherty N.G., Medina C., Harkin A. (2019). Persistent central inflammation and region specific cellular activation accompany depression- and anxiety-like behaviours during the resolution phase of experimental colitis. Brain Behav. Immun..

[bib15] Doenni V.M., Song C.M., Hill M.N., Pittman Q.J. (2017). Early-life inflammation with LPS delays fear extinction in adult rodents. Brain Behav. Immun..

[bib17] Dutta S., Beaver J., Halcomb C.J., Jasnow A.M. (2021). Dissociable roles of the nucleus accumbens core and shell subregions in the expression and extinction of conditioned fear. Neurobiol. Stress.

[bib18] Esteras N., Abramov A.Y. (2022). Nrf2 as a regulator of mitochondrial function: energy metabolism and beyond. Free Radic. Biol. Med..

[bib19] Felger J.C., Treadway M.T. (2017). Inflammation effects on motivation and motor activity: role of dopamine. Neuropsychopharmacology.

[bib20] Franklin K.B.J., Paxinos G. (2008).

[bib21] Gadotti V.M., Andonegui G., Zhang Z., M'Dahoma S., Baggio C.H., Chen L., Basso L., Altier C., MacNaughton W.K., Kubes P., Zamponi G.W. (2019). Neuroimmune responses mediate depression-related behaviors following acute colitis. iScience.

[bib22] Gebara E., Zanoletti O., Ghosal S., Grosse J., Schneider B.L., Knott G., Astori S., Sandi C. (2021). Mitofusin-2 in the nucleus accumbens regulates anxiety and depression-like behaviors through mitochondrial and neuronal actions. Biol. Psychiatry.

[bib23] Grabert K., Michoel T., Karavolos M.H., Clohisey S., Baillie J.K., Stevens M.P., Freeman T.C., Summers K.M., McColl B.W. (2016). Microglial brain region−dependent diversity and selective regional sensitivities to aging. Nat. Neurosci..

[bib24] Gruber A.J., McDonald R.J. (2012). Context, emotion, and the strategic pursuit of goals: interactions among multiple brain systems controlling motivated behavior. Front. Behav. Neurosci..

[bib26] Hart B.L. (1988). Biological basis of the behavior of sick animals. Neurosci. Biobehav. Rev..

[bib27] Hassan A.M., Jain P., Reichmann F., Mayerhofer R., Farzi A., Schuligoi R., Holzer P. (2014). Repeated predictable stress causes resilience against colitis-induced behavioral changes in mice. Front. Behav. Neurosci..

[bib28] Holder M.K., Peters N.V., Whylings J., Fields C.T., Gewirtz A.T., Chassaing B., De Vries G.J. (2019). Dietary emulsifiers consumption alters anxiety-like and social-related behaviors in mice in a sex-dependent manner. Sci. Rep..

[bib29] Hu H., Cui Y., Yang Y. (2020). Circuits and functions of the lateral habenula in health and in disease. Nat. Rev. Neurosci..

[bib31] Khor T.O., Huang M.-T., Kwon K.H., Chan J.Y., Reddy B.S., Kong A.-N. (2006). Nrf2-Deficient mice have an increased susceptibility to Dextran Sulfate Sodium–induced colitis. Cancer Res..

[bib32] Kim M.-J., Jung D.-R., Lee J.-M., Kim I., Son H., Kim E.S., Shin J.-H. (2024). Microbial dysbiosis index for assessing colitis status in mouse models: a systematic review and meta-analysis. iScience.

[bib34] Lauer J., Zhou M., Ye S., Menegas W., Schneider S., Nath T., Rahman M.M., Di Santo V., Soberanes D., Feng G., Murthy V.N., Lauder G., Dulac C., Mathis M.W., Mathis A. (2022). Multi-animal pose estimation, identification and tracking with DeepLabCut. Nat. Methods.

[bib35] Lenow J.K., Constantino S.M., Daw N.D., Phelps E.A. (2017). Chronic and acute stress promote overexploitation in serial decision making. J. Neurosci..

[bib36] Liddelow S.A., Guttenplan K.A., Clarke L.E., Bennett F.C., Bohlen C.J., Schirmer L., Bennett M.L., Münch A.E., Chung W.-S., Peterson T.C., Wilton D.K., Frouin A., Napier B.A., Panicker N., Kumar M., Buckwalter M.S., Rowitch D.H., Dawson V.L., Dawson T.M. (2017). Neurotoxic reactive astrocytes are induced by activated microglia. Nature.

[bib37] Liu L., Cheng S., Qi X., Meng P., Yang X., Pan C., Chen Y., Zhang H., Zhang Z., Zhang J., Li C., Wen Y., Jia Y., Cheng B., Zhang F. (2023). Mitochondria-wide association study observed significant interactions of mitochondrial respiratory and the inflammatory in the development of anxiety and depression. Transl. Psychiatry.

[bib38] Lubos E., Loscalzo J., Handy D.E. (2011). Glutathione Peroxidase-1 in health and disease: from molecular mechanisms to therapeutic opportunities. Antioxid. Redox Signaling.

[bib39] Lyte M., Li W., Opitz N., Gaykema R., Goehler L. (2006). Induction of anxiety-like behavior in mice during the initial stages of infection with the agent of murine colonic hyperplasia Citrobacter rodentium. Physiol. Behav..

[bib40] Mancini A., Ghiglieri V., Parnetti L., Calabresi P., Di Filippo M. (2021). Neuro-immune cross-talk in the striatum: from basal ganglia physiology to circuit dysfunction. Front. Immunol..

[bib41] Mathis A., Mamidanna P., Cury K.M., Abe T., Murthy V.N., Mathis M.W., Bethge M. (2018). DeepLabCut: markerless pose estimation of user-defined body parts with deep learning. Nat. Neurosci..

[bib42] Matisz C.E., Gruber A.J. (2022). Neuroinflammatory remodeling of the anterior cingulate cortex as a key driver of mood disorders in gastrointestinal disease and disorders. Neurosci. Biobehav. Rev..

[bib43] Matisz C.E., Lapointe V., Beekman K., Haight T., Sutherland R.J., Zovoilis A., Gruber A.J. (2025). Chronic gut inflammation differentially modulates mitochondrial and antioxidant transcriptional programs in limbic brain structures.

[bib44] Matisz C.E., Patel M., Hong N.S., McDonald R.J., Gruber A.J. (2022). Chronic gut inflammation impairs contextual control of fear. Sci. Rep..

[bib45] Matisz C.E., Vicentini F.A., Hirota S.A., Sharkey K.A., Gruber A.J. (2020). Behavioral adaptations in a relapsing mouse model of colitis. Physiol. Behav..

[bib47] Ngo V., Duennwald M.L. (2022). Nrf2 and oxidative stress: a general overview of mechanisms and implications in human disease. Antioxidants.

[bib48] Nunes N.S., Kim S., Sundby M., Chandran P., Burks S.R., Paz A.H., Frank J.A. (2018). Temporal clinical, proteomic, histological and cellular immune responses of dextran sulfate sodium-induced acute colitis. World J. Gastroenterol..

[bib49] Nyuyki K.D., Cluny N.L., Swain M.G., Sharkey K.A., Pittman Q.J. (2018). Altered brain excitability and increased anxiety in mice with experimental colitis: consideration of hyperalgesia and sex differences. Front. Behav. Neurosci..

[bib50] Ochocka N., Kaminska B. (2021). Microglia diversity in healthy and diseased brain: insights from single-cell omics. Int. J. Mol. Sci..

[bib51] Ochsner K.N., Silvers J.A., Buhle J.T. (2012). Functional imaging studies of emotion regulation: a synthetic review and evolving model of the cognitive control of emotion. Ann. N. Y. Acad. Sci..

[bib52] Okayasu I., Hatakeyama S., Yamada M., Ohkusa T., Inagaki Y., Nakaya R. (1990). A novel method in the induction of reliable experimental acute and chronic ulcerative colitis in mice. Gastroenterology.

[bib53] Pawlik R.J., Petrakova L., Cueillette A., Krawczyk K., Theysohn N., Elsenbruch S., Engler H. (2023). Inflammation shapes neural processing of interoceptive fear predictors during extinction learning in healthy humans. Brain Behav. Immun..

[bib54] Ramirez F., Moscarello J.M., LeDoux J.E., Sears R.M. (2015). Active avoidance requires a serial basal amygdala to nucleus accumbens shell circuit. J. Neurosci..

[bib55] Rezin G.T., Amboni G., Zugno A.I., Quevedo J., Streck E.L. (2009). Mitochondrial dysfunction and psychiatric disorders. Neurochem. Res..

[bib56] Rosenberg A.M., Saggar M., Monzel A.S., Devine J., Rogu P., Limoges A., Junker A., Sandi C., Mosharov E.V., Dumitriu D., Anacker C., Picard M. (2023). Brain mitochondrial diversity and network organization predict anxiety-like behavior in male mice. Nat. Commun..

[bib57] Sandberg M., Patil J., D'Angelo B., Weber S.G., Mallard C. (2014). NRF2-regulation in brain health and disease: implication of cerebral inflammation. Neuropharmacology.

[bib58] Shiba Y., Santangelo A.M., Roberts A.C. (2016). Beyond the medial regions of prefrontal cortex in the regulation of fear and anxiety. Front. Syst. Neurosci..

[bib59] Strasser A., Xin L., Gruetter R., Sandi C. (2019). Nucleus accumbens neurochemistry in human anxiety: a 7 T 1H-MRS study. Eur. Neuropsychopharmacol..

[bib60] Süß P., Hoffmann A., Rothe T., Ouyang Z., Baum W., Staszewski O., Schett G., Prinz M., Krönke G., Glass C.K., Winkler J., Schlachetzki J.C.M. (2020). Chronic peripheral inflammation causes a region-specific Myeloid response in the central nervous system. Cell Rep..

[bib61] Talley S., Valiauga R., Anderson L., Cannon A.R., Choudhry M.A., Campbell E.M. (2021). DSS-Induced inflammation in the colon drives a proinflammatory signature in the brain that is ameliorated by prophylactic treatment with the S100A9 inhibitor paquinimod. J. Neuroinflammation.

[bib62] Wager-Miller J., Murphy Green M., Shafique H., Mackie K. (2020). Collection of frozen rodent brain regions for downstream analyses. JoVE J..

[bib63] Wang J., Tu J., Cao B., Mu L., Yang X., Cong M., Ramkrishnan A.S., Chan R.H.M., Wang L., Li Y. (2017). Astrocytic l -Lactate signaling facilitates amygdala-anterior cingulate cortex synchrony and decision making in rats. Cell Rep..

[bib64] Wei D., Zhao N., Xie L., Huang B., Zhuang Z., Tang Y., Yu S., Wu Q. (2019). Electroacupuncture and moxibustion improved anxiety behavior in DSS-induced colitis mice. Gastroenterol. Res. Pract..

[bib65] Wendler E., Gaspar J.C.C., Ferreira T.L., Barbiero J.K., Andreatini R., Vital M.A.B.F., Blaha C.D., Winn P., Da Cunha C. (2014). The roles of the nucleus accumbens core, dorsomedial striatum, and dorsolateral striatum in learning: performance and extinction of Pavlovian fear-conditioned responses and instrumental avoidance responses. Neurobiol. Learn. Mem..

[bib66] Wirtz S., Popp V., Kindermann M., Gerlach K., Weigmann B., Fichtner-Feigl S., Neurath M.F. (2017). Chemically induced mouse models of acute and chronic intestinal inflammation. Nat. Protoc..

[bib67] Wu K., Liu Y., Shao S., Song W., Chen X., Dong Y., Zhang Y. (2023). The microglial innate immune receptors TREM-1 and TREM-2 in the anterior cingulate cortex (ACC) drive visceral hypersensitivity and depressive-like behaviors following DSS-induced colitis. Brain Behav. Immun..

[bib68] Xu H.-M., Huang H.-L., Liu Y.-D., Zhu J.-Q., Zhou Y.-L., Chen H.-T., Xu J., Zhao H.-L., Guo X., Shi W., Nie Y.-Q., Zhou Y.-J. (2021). Selection strategy of dextran sulfate sodium-induced acute or chronic colitis mouse models based on gut microbial profile. BMC Microbiol..

[bib69] Xu Y., Cui S.-Y., Ma Q., Shi J., Yu Y., Li J.-X., Zheng L., Zhang Y., Si J.-M., Yu Y.-C. (2018). Trans-resveratrol ameliorates stress-induced irritable bowel syndrome-like behaviors by regulation of brain-gut axis. Front. Pharmacol..

[bib70] Zelinski E.L., Hong N.S., Tyndall A.V., Halsall B., McDonald R.J. (2010). Prefrontal cortical contributions during discriminative fear conditioning, extinction, and spontaneous recovery in rats. Exp. Brain Res..

[bib71] Zhao X., Cao F., Liu Q., Li X., Xu G., Liu G., Zhang Y., Yang X., Yi S., Xu F., Fan K., Ma J. (2019). Behavioral, inflammatory and neurochemical disturbances in LPS and UCMS-induced mouse models of depression. Behav. Brain Res..

[bib72] Zheng J., Sun Q., Zhang M., Liu C., Su Q., Zhang L., Xu Z., Lu W., Ching J., Tang W., Cheung C.P., Hamilton A.L., Wilson O'Brien A.L., Wei S.C., Bernstein C.N., Rubin D.T., Chang E.B., Morrison M., Kamm M.A. (2024). Noninvasive, microbiome-based diagnosis of inflammatory bowel disease. Nat. Med..

[bib73] Zheng J.-Y., Li X.-X., Lin W.-Y., Su S., Wu H.-C., Hu R.-D., Pan H.-F., Ye J.-H., Cai Y.-F., Zhang S.-J. (2023). Huang-Lian-Jie-Du decoction alleviates depressive-like behaviors in dextran sulfate sodium-induced colitis mice via Trem2/Dap12 pathway. J. Ethnopharmacol..

[bib74] Zonis S., Pechnick R.N., Ljubimov V.A., Mahgerefteh M., Wawrowsky K., Michelsen K.S., Chesnokova V. (2015). Chronic intestinal inflammation alters hippocampal neurogenesis. J. Neuroinflammation.

