# Supplementary material for: Chronic gut inflammation potentiates place aversion in intrinsic and conditioned contexts
Source: Brain Behav Immun Health. 2026 Oct 8;58:101383. doi: 10.1016/j.bbih.2026.101383 (PMC13685889; doi:10.1016/j.bbih.2026.101383)
Supplement: Supplementary file 1 [file mmc1.docx]

**Supplementary material**

**Supplementary Figure 1. Relationship between centre dwell time and fecal LCN2 in control mice.**

Correlation between time spent in the centre of CPA apparatus and fecal LCN2 among vehicle-treated animals. Data are mean ± SEM; ; n=9 control male mice.

**Supplementary Figure 2.**

Total time freezing in paired and unpaired contexts during discriminative fear to context testing at recent (A) and remote (B) time point. (C) The difference in proportion of time freezing in the unpaired context at the remote time point; one sample t-test to theoretical mean of 0. Data are mean ± SEM; n=9-12 male mice per treatment group. *p<0.05.

**Supplementary Figure 3. Expression of mRNA transcripts in the ACC and NAc of control mice.**

(A) Mean expression of (A) *Nrf2* (B) *Mt-Co1* (C) *Il1b* and (D) *Gpx1*, using *Hprt* as a reference gene, in the ACC and NAc. Data represent mean ± SEM; n=6. * p<0.05, ** p<0.01. ***p<0.001.
